# Condensed trajectory of the temporal correlation of diseases and mortality extracted from over 300,000 patients in hospitals

**DOI:** 10.1371/journal.pone.0257894

**Published:** 2021-10-05

**Authors:** Hyojung Paik, Jimin Kim

**Affiliations:** 1 Division of National Supercomputing, Center for Supercomputing Applications, Korea Institute of Science and Technology Information, Daejeon, Republic of Korea; 2 Department of Data and HPC Science, University of Science and Technology, Daejeon, Republic of Korea; University of Salamanca, SPAIN

## Abstract

Understanding mortality, derived from debilitations consisting of multiple diseases, is crucial for patient stratification. Here, in systematic fashion, we report comprehensive mortality data that map the temporal correlation of diseases that tend toward deaths in hospitals. We used a *mortality trajectory* model that represents the temporal ordering of disease appearance, with strong correlations, that terminated in fatal outcomes from one initial diagnosis in a set of patients throughout multiple admissions. Based on longitudinal healthcare records of 10.4 million patients from over 350 hospitals, we profiled 300 mortality trajectories, starting from 118 diseases, in 311,309 patients. Three-quarters (75%) of 59,794 end-stage patients and their deaths accrued throughout 160,360 multiple disease appearances in a short-term period (<4 years, 3.5 diseases per patient). This overlooked and substantial heterogeneity of disease patients and outcomes in the real world is unraveled in our trajectory map at the disease-wide level. For example, the converged dead-end in our trajectory map presents an extreme diversity of sepsis patients based on 43 prior diseases, including lymphoma and cardiac diseases. The trajectories involving the largest number of deaths for each age group highlight the essential predisposing diseases, such as acute myocardial infarction and liver cirrhosis, which lead to over 14,000 deaths. In conclusion, the deciphering of the debilitation processes of patients, consisting of the temporal correlations of diseases that tend towards hospital death at a population-wide level is feasible.

## Introduction

Understanding which clinical risks lead to fatal consequences, such as prognosis variations among cardiac patients based on comorbidities, is a key component for the establishment of risk stratifications and health policy [1–3]. Data-driven approaches using large-scale medical records for non-research purposes have demonstrated the validity of establishing correlated diseases, including patterns in the timing of each disease’s appearance [4–7]. However, unraveling mortality patterns with fatal outcomes, which belongs to the diagnostic timeline, is indispensable and directly helps clinical care and healthcare strategies in the population. Heretofore, the landscape of the temporal appearance of diseases leading to hospital deaths in populations has not been actively established. A recent reported study was conducted using data from 7.2 million patients to create the Danish Disease Trajectory Browser (DTB), which could identify diagnostic pairs with statistically significant directionality and explore disease progression patterns [8].

Here, we used large-scale healthcare records, consisting of diagnosis records and fatal outcomes, to provide a comprehensive map of mortality-associated disease patterns in real clinical boards. To identify mortality trajectories across all diseases in multiple hospitals in the US, we analyzed population-wide administrative healthcare records using the Healthcare Cost and Utilization Project (HCUP) [9]. HCUP is the comprehensive source of national hospital data in the US, used to study health care delivery and patient outcomes over time at the national, regional, state, and community levels. HCUP has released State Inpatient Databases (SID) as encounter-level longitudinal records (over 20 years of hospital records). We used the SID for California (SIDCA), which contains data for over 10.4 million hospitalized patients from over 350 non-federal hospitals in California. These records contain no direct patient identifiers and are publicly available for use upon submission of a data use application. In our previous work, using the identical data resource, we highlighted the identification of unknown risk of schizophrenia patients, and discovered the association between schizophrenia and rhabdomyolysis, a rare muscle disease from the disease trajectories [10].

We performed a systematic investigation to assess *mortality trajectories* in these records. By this term, we mean the sequential patterns of disease preceding to death that were shared among patients across California hospitals. The sequences in each mortality trajectory include subsequent diagnoses for each admission (< 1 year and FDR <0.1 of temporal correlations of diseases) and associated fatal outcomes. We identified 300 trajectories with strong temporal directionality and statistical significance that thereby yield a global view of the most populated, directional co-morbidities and fatal outcomes observed in the US in California hospitals. This study presents explicit pathways from the initial diagnosis records to the final ones that tend toward fatal outcomes in hospitals at a population level. The data analyses presented here are useful for healthcare strategy and policy, as they exhibit fatal outcomes in a corresponding trajectory and are thus amenable to stratify patients by mortality and associated diseases.

From the data, we found that the proportion of hospital deaths and diseases accrued from multiple admissions for significantly correlated disease appearances is considerable. In three major mortality trajectories involving the largest number of fatal outcomes, we highlighted key diagnoses that tend toward over 14,000 fatal consequences throughout sequential diseases for each age group. Thus, our findings can be used to define groups of patients to include in prognosis research studies and diverse cohort studies. Our analyses show the importance of stratifying a cohort by preceding diseases to understand the heterogeneity of disease prognosis in clinics.

## Materials and methods

### Population-wide administrative healthcare records

The data used in this analysis was obtained from the California set of HCUP, http://www.hcup-us.ahrq.gov/), called SIDCA (State Inpatient Database, California). This database contains de-identified admission and discharge information for >350 community hospitals in California. These include nonfederal, general, other specialty hospitals, and academic medical centers. It excludes non-community hospitals, such as federal hospitals (e.g., Veterans Affairs), long-term care hospitals, and clinical units within institutions (e.g., prisons). For each hospitalized patient, the database contains up to 25 diagnosis codes by chart order using the International Classification of Disease, Ninth Revision, Clinical Modification (ICD-9-CM).

We assumed that the first reported diagnosis code reflected the primary diagnosis for each hospital stay. Because SIDCA contains a unique identifier for every individual, we were able to identify readmissions for the same patients over time and across hospitals. We merged five SIDCA versions, which were generated annually (2006 to 2010). Each of the five SIDCA datasets involves accumulated records covering up to over 20 years in a longitudinal manner. While each SIDCA version involved unique identifiers for each patient, meta-mapping of the patient identifiers across data versions is not available. To prevent data redundancy in the merged SIDCA data set, we used records of only deceased individuals and their hospitalization records in 2006–2009 generated SIDCA dataset, except for the latest version of the SIDCA generated in 2010.

In this study, all diagnosis codes were rounded to the 3-digit code level (a 3-digit code providing a general description of a disease, such as “250” for diabetes) to preserve accuracy [11] and minimal overlap of diagnoses; 250.41 for diabetes with renal manifestations, juvenile type is a subclass of diabetes.

### Statistical significance of the temporal correlation of disease

To determine the disease correlation in time-directional order, the method of our previous attempts was used [10]. In summary, we used relative risk (RR) measurement to quantify the occurrence of disease pairs (*Disease i–Disease j*) within 1 year in a patient [12]. When RR > 1, the co-occurrence of the two diseases was higher than that expected for diseases co-occurring by random chance. Then, we quantified the likelihood that one disease would occur before or after another (*δ*_i→j_ for *Disease i → Disease j*) using the dates of admissions associated with two diseases in each patient [4]. To calculate *δ*_i→j_, we begin counting date differences between when disease *i* was diagnosed before disease *j* in patient *p* and represent this number as *d*^*p*^_*i→j*_ (*d* ^*p*^_*i→j*_ = *sign* (date of admission for disease *j* in patient *p*–date of admission for disease *i* in patient *p*), where *sign* stands for the *Signum* function, *d* ^*p*^_*i→j*_ = [-1,1]). Multiple re-diagnoses or re-hospitalizations for the same disease in a patient *p* were ignored, and only the initial date of admission for a disease was used as a date of diagnosis for disease *i* or disease *j* to determine the timing of each disease in a pair for patient *p* (*d* ^*p*^_*i→j*_). In addition, we only counted *d* ^*p*^_*i→j*_ when the length of duration between dates of admissions for disease *j* and disease *i* was less than 1 year. A value of *d* ^*p*^_*i→j*_ > 0 indicates the following: an initial admission for disease *i* occurred before the first admission for disease *j* in a patient *p* within one year. Then, the value of *δ*_i→j_ was determined by the mean value of *d* ^*p*^_*i→j*_ among the set of patients who were diagnosed with diseases *i* and *j* in one year. Thus, a value of *δ*_i→j_ > 0 indicates that over half of admissions for disease *i* occurred before the admissions for disease *j* by one year among the patients who were diagnosed as both of these diseases. Alternatively, a value of *δ*_i→j_ < 0 denotes the opposite. The statistical significance of co-occurrences (RR) and the temporal directionality of diseases (*δ*_i→j_) were determined using a binomial test (Benjamini–Hochberg FDR < 0.1) [7]. Finally, we used pairs of correlated diseases with time directionality whose mathematical relationships were statistically significant (RR > 1, FDR < 0.1; *δ*_i→j_ ≠ 0, FDR < 0.1) for further analysis. We use the term *temporal correlation* to describe this relationship.

### Defining one mortality trajectory and clusters of trajectories

Based on patients sharing two pairs of temporally correlated diseases (*Disease 1 → 2* and *Disease 2 → 3*), we joined multiple disease-to-disease correlations by concatenating two pairs of sequential disease diagnoses into three or more steps of overall disease occurrences among patients (*Disease 1 → 2 → 3*) [7]. A greedy algorithm was used to find further steps in disease paths that encompassed more patients. Disease pairs were sorted in descending order according to patient counts. Pairs with an overlapping diagnosis were found starting from the top of the list, and the number of patients following the full trajectory to death was counted. We stopped when a trajectory had no patients following it.

## Results

### Study set and patient characteristics

To build mortality trajectories, we needed sufficiently broad and longitudinal health records within a unified format and without redundancy of records. We used the California SID data set (SIDCA), which contains inpatient records, ICD-9-CM diagnosis codes, and patient outcomes for each hospitalization across >350 community hospitals in California (mean number of hospitals = 358.6 ± 4.4: 354 hospitals in the SIDCA built at 2010, 354 hospitals in the 2009 version, 361 hospitals in the 2008 version, 360 hospitals in the 2007 version and 364 hospitals in the 2010 version).

**Fig 1:**
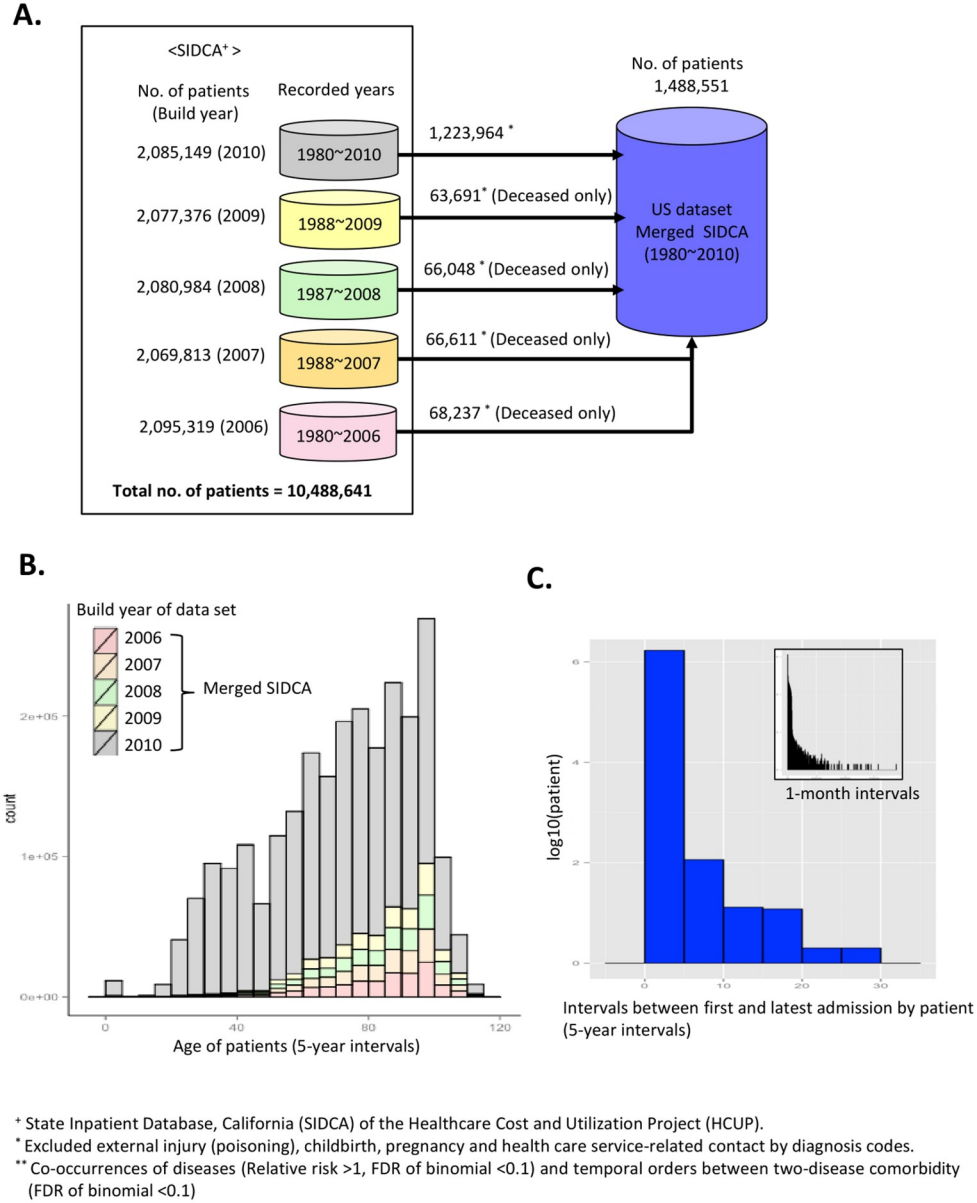
Overview of study set. (A) Overview of the data set build. We prepared the study data set by combining five SIDCA data sets, which were released annually (2006–2010). In summary, the merged SIDCA covers longitudinal records (1980–2010) for >1.4 million patients without data redundancy. (B) Age distribution of the merged SIDCA. Each color represents the build year of that data set. (C) Distribution of time intervals (Data are presented as mean) between the first and last admission for each individual. Based on the first and latest admissions for each individual, we calculated the traced dates. The merged SIDCA represents mainly the disease progression for 1.4 million adult patients within 5 years, leading to 290,253 hospital deaths in the US.

The first diagnosis code in a patient’s chart was used as the primary disease for each hospital stay. Each patient had a unique identification code, making it possible to detect readmission of the same patient in a longitudinal manner across hospitals. We used ICD-9-CM diagnosis codes to filter out records for non-disease conditions, such as diagnosis chapters referring to injuries, obstetrics, and healthcare-related contacts as defined in ICD-9-CM chapters (Fig 1A). Because the SIDCA data sets are built and released annually, we independently selected records of deceased patients in early data sets and subsequently added later data releases. This process minimized redundancy of records in the merged SIDCA (Fig 1A).

The merged SIDCA study set covered 2,272,018 hospitalizations of 1,488,551 individuals from all non-federal hospitals in California, and 290,253 death outcomes (S1 Table). As depicted in Fig 1B, most of the merged SIDCA data were drawn from the latest version (build year 2010, gray bar in Fig 1B), and covered the filtered set from the 2006 to 2009 versions. Patient age in the merged SIDCA had a skewed distribution from middle age (40s) to old age (80s). Pediatric groups were excluded. The mean age of patients in the data (merged SIDCA) was 63.77 ± 19.58. Of this group, 46.4% were male, 52.4% were female, and 1.2% were unknown (S1 Table). Fig 1C shows power-law distributions of observation times for each individual in the merged SIDCA data, based on the length of time between the first and last admission dates. Most of the merged SIDCA data included diagnostic timelines for each patient for 4 years (median interval between first and last admission dates of a patient = 40.58 months ± 2.5 months) (Fig 1C). The longest duration between the first and most recent admission dates in a patient was 26 years. Thus, the merged SIDCA data represent diagnosis timelines of 1.4 million adults leading to 290,253 hospital deaths from 1980 to 2010 (19.5% of patients).

### Tracing patients from the initial disease diagnosis to fatal outcome in the clinics

In each of the mortality trajectories, the sequence followed by a prior disease condition included subsequent diagnoses in different admissions in one year, with strong correlation and temporal directionality. Thus, a pair of temporally correlated diseases is a building block of a trajectory. As noted in the Methods section, we used the relative risk (RR) measurement and the date of admission for a disease to identify the occurrence of two diseases in one year in one patient and the time spans between the diagnoses. This process allowed us to define the temporal order of disease diagnoses that occurred more frequently than expected by chance [4,12]. The statistical significance of the identified order of disease-associated admissions and co-occurrence of diseases in a patient was determined using a binomial test (FDR < 0.1) [7]. To model this process for near-term disease appearances, we considered only disease pairs that occurred in the same patient within one year. Of the 691 diseases in the merged SIDCA (S1 Table), 168 diseases were associated with at least one temporally aligned comorbid disease (FDR<0.1, S2 Table).

We then re-constructed diagnosis timelines of patients by combining these temporally correlated disease pairs (number of disease pair with significant temporal correlations = 300). We built trajectories that started with all possible diseases using a greedy algorithm that identified major mortality timelines in patients. Subgroups of subsequent diagnoses after the selected initial disease pair or non-significant disease diagnoses in following steps were omitted. By individually iterating over each starting disease state, we mapped the details of 300 trajectories on each timeline from initial diagnosis through intermediate states to death. In total, the mortality trajectories started with 118 diseases and 311,309 patients, went through 175,556 distinct disease-to-disease transitions, and reached 59,794 fatal outcomes.

**Fig 2:**
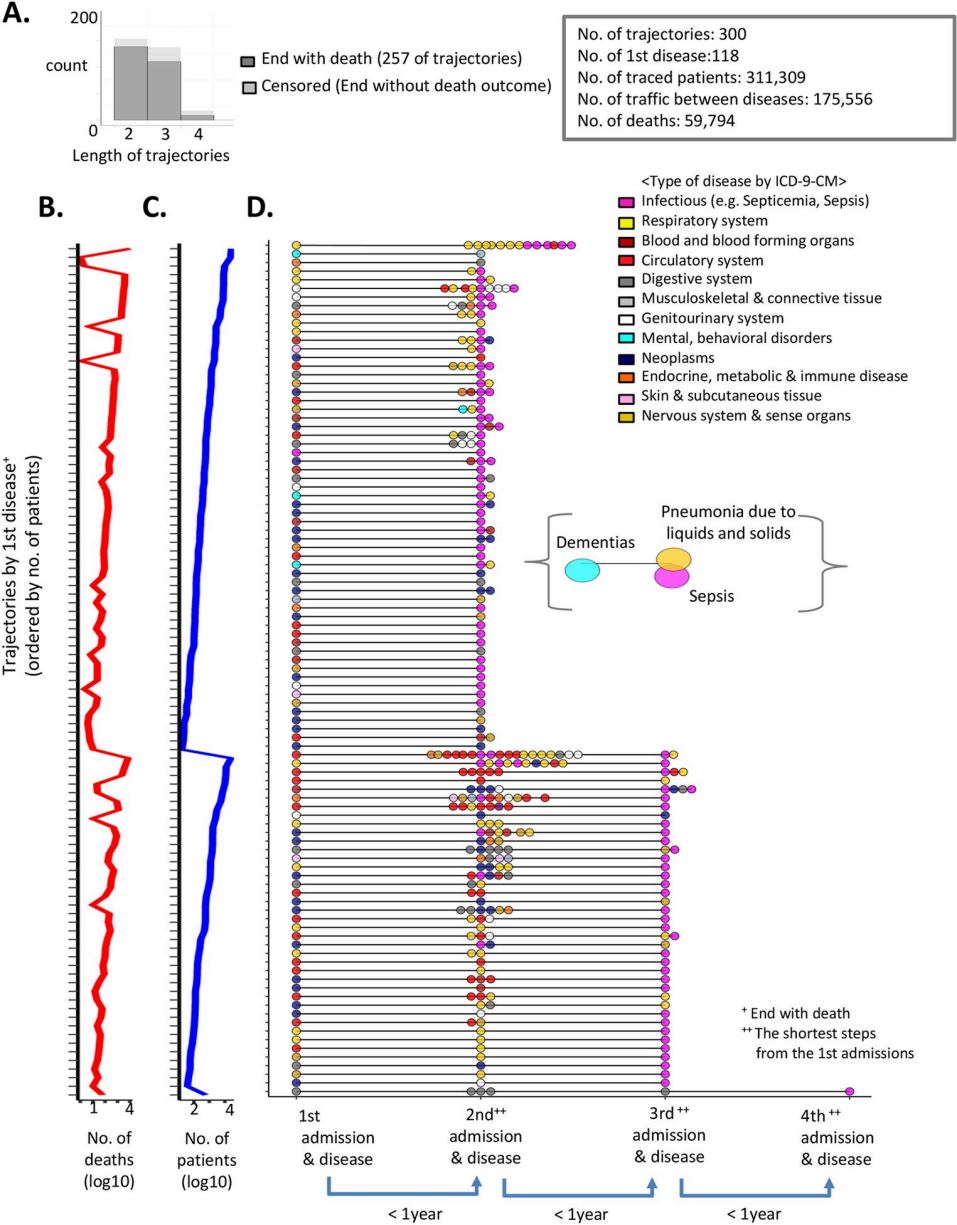
Scaffold map of mortality trajectories in the US hospitals. (A) Distribution of the identified trajectory lengths. The dark gray bar represents trajectories with fatal outcomes recorded as the latest disease progressions. The light gray bars denote trajectories with censored fatal outcomes. (B-D) We present the overview of 257 trajectories among 300 total identified trajectories (the dark bars in Fig 2A). (C) In the y-axis, the trajectories are arranged according to the total number of deaths in descending order. The x-axis represents the total number of deaths in log10 scale. (B) This plot shows the number of deaths for each trajectory by the identical order in Fig 2C. (D) An aligned view of disease steps by following the admissions for the diseases within each trajectory. The y-axis denotes the drawn trajectories by the identical order in Fig 2C. The x-axis represents each admission step for the correlated diseases. Each node color denotes the type of disease as determined using the ICD-9-CM codes.

Of the 300 trajectories, the longest mortality trajectory had four of admissions steps by sequential disease appearances. In 257 trajectories, death outcomes occurred at the final condition in the trajectory (Fig 2A, dark gray bar), while in 43, the traced patients were still alive, or there were no death outcomes at the final disease diagnosed (Fig 2A, light gray bar).

### The population-wide trajectory map quantifies the heterogeneity of the disease patients

Based on the established trajectory map, we investigated the patterns of diseases that precede to fatal consequences, such as the conserved temporal sequence of diseases among the mortality pathways and degree of diversity within the disease patients. We aligned the non-truncated 257 trajectories that terminated with death outcomes at the latest disease and sorted them by the number of patients (Fig 2C) and deaths (Fig 2B). As shown in Fig 2D, in the x-axis, each circle node represents each subsequent disease step in each trajectory, which is strongly correlated with prior diseases within 1 year. The y-axis denotes trajectories for each initial disease with the same order of Fig 2B and 2C. The varied colors of circle nodes visualize the overall disease trends in each step for each trajectory based on ICD-9-CM codes for a circle node (Fig 2D). The trajectory in brackets denotes one example, which started with dementia. The ICD-9-CM dementia code is categorized as a mental disorder, which filled the circle as cyan color. In the second admission (< 1 year from the first), the dementia patients were diagnosed with either sepsis (infectious diseases in magenta) or aspiration pneumonia (i.e., pneumonia due to liquid and solid, respiratory diseases in yellow). As a result, for the first time, we profiled the landscape of mortality trajectories for 287,118 patients and 58,257 deaths from 100 initial diseases via strongly correlated interim diseases and ranked them by mortality.

The initial and interim disease nodes were heterogeneous. Nevertheless, the majority of dead-ends converged into either sepsis (magenta circles for infectious diseases) or pneumonia (yellow nodes for respiratory diseases; Fig 2D). In addition, none of the identified trajectories started with sepsis. The global view of the trajectory map indicates that sepsis is a predominant dead-end stage in hospitals and a common consequence mediated by the debilitation process of patients consisting of sequentially correlated diseases. Thus, sepsis is a non-sporadic disease, and we identified 43 prior diseases of sepsis, including an interesting association between malignant neoplasms of lymphoid and sepsis (ICD-9 code = 202, “other malignant neoplasms of lymphoid and histiocytic tissue”). Without the evidence of time directionality between lymphoma and sepsis, the co-occurrence of sepsis among cancer patients has been reported in an earlier national-wide study [13]. Our analysis presents an explicit pathway from cancer to sepsis that has progressed within a short time span with a furcate pathway (the mean time interval = 83.17 ± 61.99 days; the mean age of patients = 66.95, S3 Table).

Including sepsis (an extreme case), a substantial proportion of diverse interim diseases preceding the end stage manifested that the heterogeneity of disease patients in the real world is prevalent. From our map, patients underwent 175,556 multiple hospitalizations in a series of correlated diseases, and then patients reached the fatal outcomes. Out of 59,794 disease patients and associated fatal outcomes, 74.5% (44,598) accrued throughout 160,360 multiple disease appearances in a short-term period (<4 years, 3.5 diseases per patient). For example, osteomyelitis periostitis patients (i.e., inflammation of bone, ICD-9 code: 730) were stratified based on the correlated previous diagnoses, cellulitis and diabetes (S3 Table). The subgroup of patients, osteomyelitis patients with diabetes, were likely to be in polypharmacy states, and also extended to heterogeneity in treatment responses due to glucose-induced proinflammatory cytokines [14]. By suggesting the heterogeneity of diseases based on correlated pre- or post-diseases, our results facilitated the adequate stratification of patients for cohort studies.

Moreover, our offers a clustered view of mortality trajectories by overlaying them by shared diagnoses and patterns of disease-to-disease progressions [7,15]. A total of 16 clusters comprised the landscape of a set of associated trajectories, including Cluster 12, a merged set of trajectories that comprise chronic obstructive pulmonary diseases (COPDs), and Cluster 7, a set of cancers and metastasis patterns, such as colorectal lung metastasis among elderly patients [16] (S2 Fig).

In summary, we provide a comprehensive and widely applicable model that can be useful for risk stratification of patients by displaying confounding factors, such as predispositions and future disease patterns of patients.

### Prioritization of trajectories using the number of fatal outcomes

In addition to providing an important analysis of temporal disease associations at the population scale, the fatal outcome of a disease in our model facilitates the ranking of risk by the number of fatal outcomes in each mortality trajectory. Thus, we prioritized the key preceding diagnoses associated with subsequent diseases and substantial numbers of fatal outcomes. We ranked the deadliest trajectories by the total number of deaths in each trajectory, and then selected the deadliest ones for each age group based on the mean age of patients in each trajectory, including younger patients (mean age, <60 years), moderately older patients (mean age, ≥60 and ≤75 years), and elderly patients (mean age, >75 years). We visualized the trajectories as connected paths through nodes for diseases (circle nodes) that terminated in death (square nodes). We drew a line (i.e., edge) between diseases (circle nodes) and fatal outcomes (square nodes) to signify the transition of patients in each step of the traced trajectory. Edge colors and widths signify the mean age and number of traced patients in the disease and death nodes, respectively.

**Fig 3:**
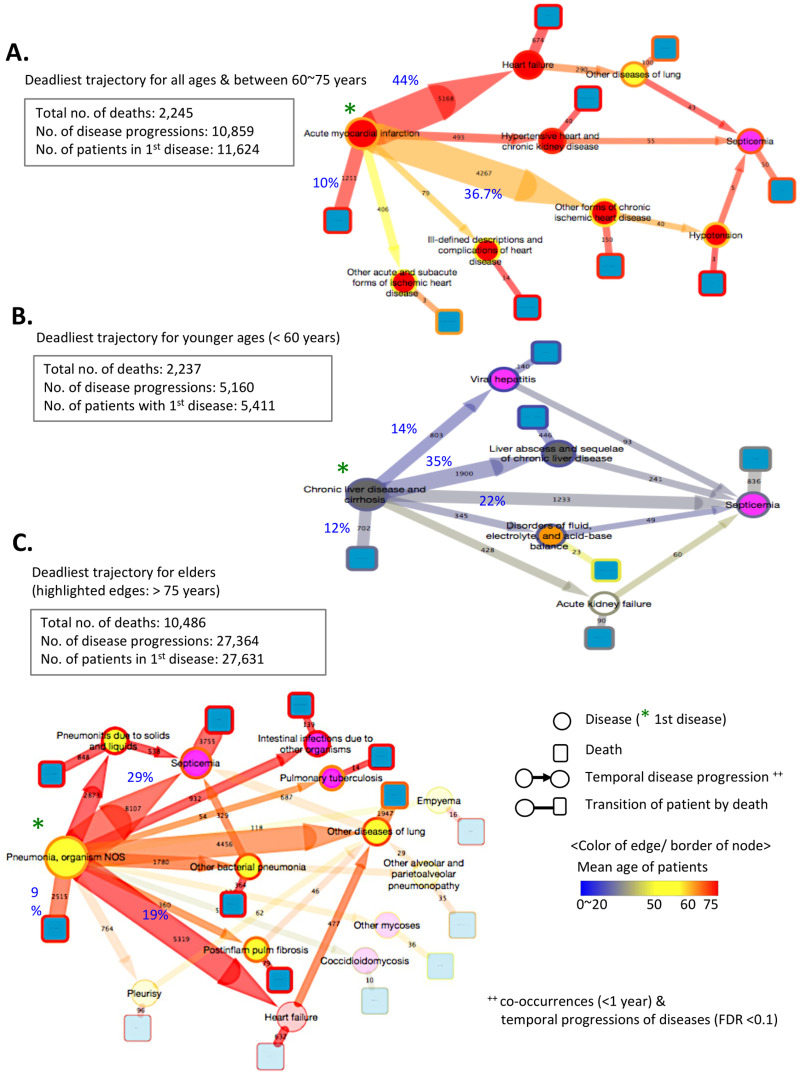
The deadliest trajectory. The deadliest trajectory in California, based on the number of associated deaths according to age group: (A) all ages and moderately aged (60–75 years) group; (B) younger group (mean age, <60 years); and (C) elderly age group (mean age, >75 years). The pattern for the moderately aged group was similar to the overall pattern in (A).

The selected trajectories present the essential predisposing diseases in each life-cycle phase, which led to over 14,000 deaths and other subsequent diseases, such as liver cirrhosis for younger patients, pneumonia for elders and acute myocardial infarction (AMI) for overall groups. The deadliest trajectory in all age groups (and mid age groups) started with acute myocardial infarction (AMI; 11,624 patients) (Fig 3A). After the diagnosis of AMI, 4,267 of the 11,624 patients (36.7%) were diagnosed with ischemic heart disease by the next admission (mean age, 68.12 years). However, 44% of elderly patients (5,166 patients) were diagnosed with heart failure within 1 year (mean age, 75.01 years). Of note, the traced subsequent diseases of AMI in elderly patients showed serious outcomes, with higher case fatality ratios (CFR) in heart failure (0.15 = 674/5168), whereas the younger patient group was transferred to ischemic heart disease, resulting in lower CFR (0.05 = 250/4267). Interestingly, a total of 103 surviving patients from the second or third comorbid disease were eventually hospitalized with sepsis, which led to death in half of them. Although there are diverse confounding factors, such as undetected infections, it is an opposite order to the known pattern, sepsis-induced cardiomyopathy [17]. Otherwise, among the younger patients (mean age <60 years), the deadliest trajectory started with chronic liver diseases and cirrhosis (Fig 3B). Approximately 31% of patients (1,678 of 5,411) followed mortality pathways that spanned diverse diseases, leading to 836 deaths from sepsis (Fig 3B). Fig 3C depicts the trajectory starting with pneumonia in 27,631 patients, which accounted for the most deaths in elderly patients (10,486 fatal outcomes). Patients developed a distinct series of diseases after pneumonia, including heart failure (19% of pneumonia patients) or sepsis (29%), by the next admission.

Owing to the diverse subsequent diagnoses and death outcomes after the initial presentation of disease, our trajectory model allowed us to prioritize key diagnoses that can precede to fatal consequences and can thus be used to define groups of patients to include in cohort-based studies, including prevention studies for health policy and clinical trials.

## Discussion

This study was an extensive temporal analysis of disease-to-disease comorbidity relationships for >600 diseases in 10.4 million patients and 290,253 fatal outcomes in California, US hospitals. In total, we identified 300 mortality trajectories beginning with 118 diseases in 311,309 patients. They consequent to 59,794 fatal outcomes throughout 175,556 of disease-to-disease transitions by distinct admissions. For the first time, our approach presented the time-aligned patterns of diagnoses that led to fatal outcomes in the hospital by leveraging large-scale healthcare records, which were routinely gathered for billings. A substantial proportion of re-hospitalization for sequential diagnoses and associated fatal consequences showed the validity of our trajectory approach to stratifying a disease cohort into the traced disease patterns and prognosis to understand the heterogeneity of disease patients in a time-dimensional space.

Several limitations of the study should be noted. First, health records are not intended specifically for research, as the codes may not be accurate. Our data captures only inpatient records in the hospitals; outpatient diseases, such as common flu, and fatal outcomes after the discharge of patients are invisible in our dataset, including home deaths and deaths in hospice (e.g., cancer deaths) [18]. A nationwide study describing participants’ primary, multimorbid and outpatient diseases from birth to death would be ideal. However, practical constraints make such a large study unfeasible. In addition, we used the Greedy algorithm to find further steps in disease paths that encompassed more patients and to identify major mortality timelines. The algorithm is known to have limitations because it determines the optimal path selection for that moment at every step, regardless of the overall information. Taken as a whole, it has the downside of not being able to guarantee that the path selection is optimal at each step.

Tracked sequences in each mortality trajectory included subsequent diagnoses and co-occurred fatal outcomes by re-admissions among a selected initial disease and patients. Here, we note that the main cause of deaths are retrospectively determined by a medical examiner regarding the overall condition of patients, then reported to the death registry of US. As we depicted, we’ve conjugated co-occurred fatal outcomes to disease diagnoses without consideration of causality. Therefore, our trajectory model shows the propensity of diageneses sequences to the death outcome in clinic. Although the records of death registry present the main cause of death, such as heart failure, detailed progressions are absent. Adequate stratification of patients preceding fatal outcome is a premise of tailored care of patients. Thus, the unique advantage of our approach over traditional statistical analysis is its ability to map the debilitation course resulting in deaths and to stratify patients into distinct groups according to disease patterns related to mortality.

We acknowledge that non-disease associated cause of death are not involved in our model, such as severe trauma. However, the majority of the injury-associated hospitalization are conducted via the visit of Emergency Room (ER). Owing to the truncation of those ER visits in our SIDCA, we focused disease associated mortality trajectory in our study. For the modeling of diagnosis trajectory from external trauma, an ER visit data, such as SEDD (the State Emergency Department Database) of HCUP (https://www.hcup-us.ahrq.gov/seddoverview.jsp) would be analyzed in further study. Altogether, we were able to rank trajectories based on the accrued mortality and suggest the illness worsening course resulting in hospital deaths. This helps to conceptualize patterns of clinical timelines and mortalities of patients who were diagnosed with a correlated disease.

Comparing other previous work [8] (*Danish Disease Trajectory Browser*; http://dtb.cpr.ku.dk/), the originality of our study is that our trajectory model traced a fast disease progression within 1-year intervals among more severe diseases. For in-depth comparison of our results and other previous attempts, we presented all the detail of the modeled trajectory of ours in the S3 Table.

For the clinical inference of each mortality trajectory, it is essential to assess to what extent the directionality reflects underlying causal patterns in a hospital. For example, it is interesting to deduce whether the AMI (Acute Myocardial Infarction) is the cause, or whether AMI is a surrogate disease of other confounding factors for the deaths from sepsis-induced cardiac dysfunctions. Including an interesting disease correlation (i.e., lymphoma), our analysis suggests the major prior diseases of sepsis by each age group within a short time span (< 3 years), such as cirrhosis for young patients, pneumonia for elders and AMI for the middle or overall group. The presented global picture of sepsis manifests the presumed underlying mechanism of heart failure in sepsis, such as abnormal cytokine release under lymphoma and cirrhosis states among sepsis [19,20]. It is supportive that AMI is a pre-existing disease of sepsis patients, which might be associated with myocardial depressant factor for the cardiac dysfunction of sepsis [17,21]. The ability to make data-supported inferences of disease mortality (inflammation of cirrhosis associated with sepsis) and of medical systems issues (under-recognition of co-morbidity of AMI in sepsis) demonstrates the power of the trajectory analysis. For decades, numbers of possible mechanisms have been independently proposed based on the limited scale of the population [22–24]. A study proposed in 2016 used relatively large-scale data from 6.6 million patients to identify trajectories that significantly altered sepsis mortality. The authors found an increase in sepsis mortality from key starting points such as alcohol abuse, diabetes, cardiovascular diagnosis and cancer in the sepsis network [25].

This study presents a large-scale examination of the temporal pattern of death in hospitals from the initial presentation of disease in the records across the diverse disease spectrum by tracking millions of healthcare records. The insight gained from this study may promote clinical outcomes that benefit from considering the most probable next step in disease progression, including fatality and the heterogeneity of prior chronic or acute diseases. Owing to the direct use of the health records from hospitals, a major prospective application in using the trajectories established here is the stratification of patients for precision medicine by combining them with the molecular signatures of each patient, for example via whole-exome sequencing, for better disease prognosis of individual patients along the course each patient will take.

## Supporting information

S1 Fig Dynamic visualization of all mortality trajectories in the US. This figure presents the captured images from the dynamic visualization of all mortality trajectories (https://www.youtube.com/watch?v=jJMds31-e2g). Sequential presentations of disease nodes were determined according to mean age of patients at disease incidence. We traced 311,309 patients. Interestingly, 38.1% of fatal outcomes involved septicemia via diverse disease progressions in the hospitals (green box).
(PDF)

S2 Fig Cluster of trajectories. We offers a clustered view of mortality trajectories by overlaid them by shared diagnoses and patterns of disease-to-disease progressions. (A-B) Of total 16 clusters, we present the first and second largest clusters. (A) The largest cluster, Cluster 12, covered disease patterns for >90,000 patients who had developed chronic obstructive pulmonary disease (COPD) and other circulatory heart diseases. (B) Cluster 7 depicts cancer and metastasis by tracking 17,781 patients and 1,566 deaths. Death nodes are hidden to improve the visibility of the clusters.
(PDF)

S1 Table Data statistics of the State Inpatient Database of California (SIDCA). Data resource of the Healthcare Cost and Utilization Project (HCUP) covering 97% of hospitals in the USA. ^1^Years of data set generations. Merged data set covers up to ~26.1 years of longitudinal events for a patient counted by administration month. For each inpatient event, up to 25 diagnosis codes were assigned. ^2^Covered years of records by build year versions. ^3^Excluded by diagnosis chapters for injury, symptom, childbirth, pregnancy, and healthcare service.
(DOCX)

S2 Table List of temporally correlated disease pairs. (XLSX)

S3 Table Data summary of traced disease trajectories. (XLSX)
