# Supplementary material for: Condensed trajectory of the temporal correlation of diseases and mortality extracted from over 300,000 patients in hospitals
Source: PLoS One. 2021 Oct 5;16(10):e0257894. doi: 10.1371/journal.pone.0257894 (PMC8491897; doi:10.1371/journal.pone.0257894)
Supplement: S1 Fig — This figure presents the captured images from the dynamic visualization of all mortality trajectories (https://www.youtube.com/watch?v=jJMds31-e2g). Sequential presentations of disease nodes were determined according to mean age of patients at disease incidence. We traced 311,309 patients. Interestingly, 38.1% of fatal outcomes involved septicemia via diverse disease progressions in the hospitals (green box). (PDF) [file pone.0257894.s001.pdf]

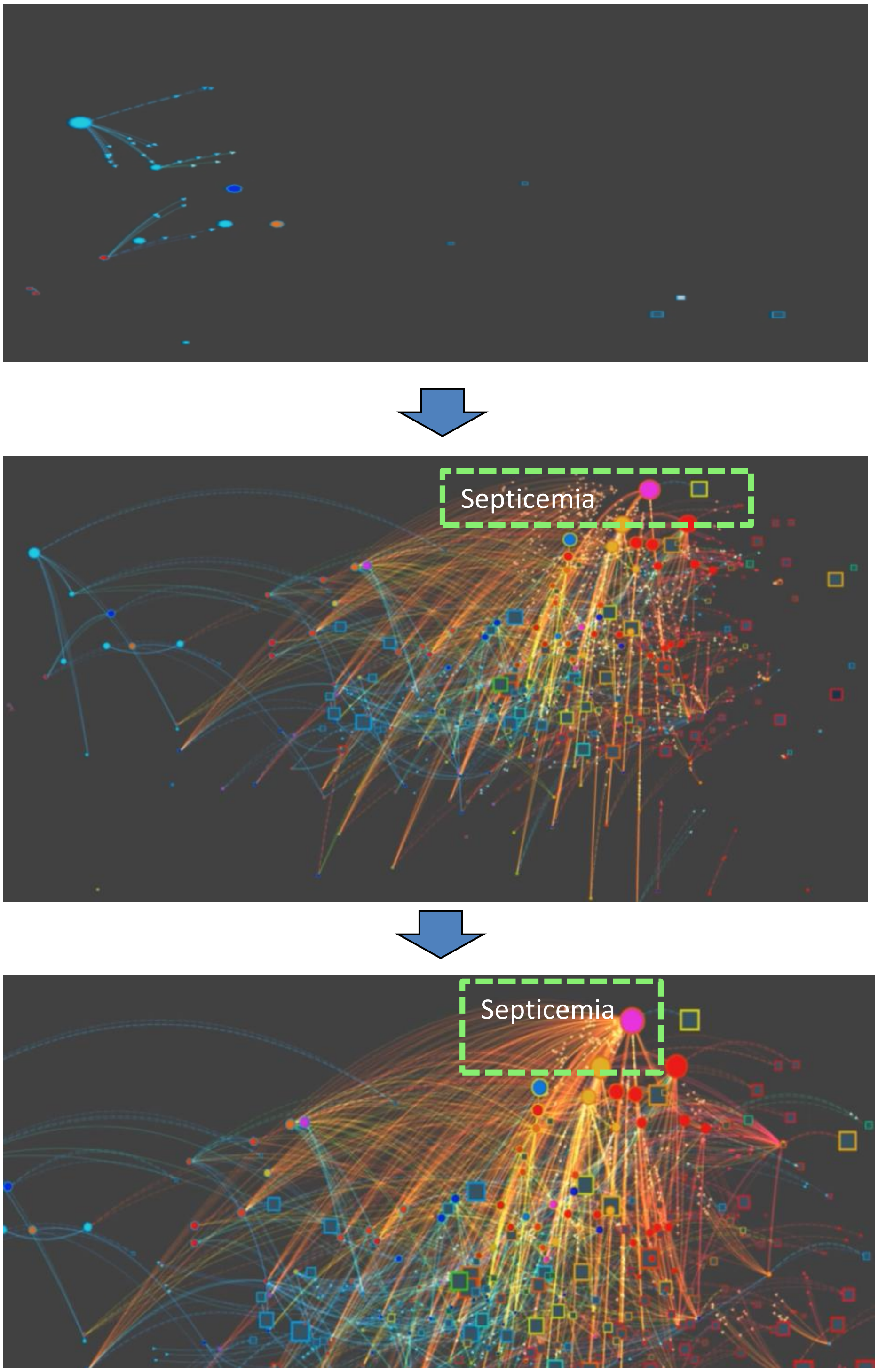

Mean age 54

Mean age 83

Mean age 91

No. of traced patients: 311,309
No. of traffic between diseases: 175,556
No. of deaths: 59,794 (death with septicemia = 22,807)

Total play time: 32.0 sec

(Optimized in Chrome)

Supplemental figure 1. Dynamic visualization of all mortality trajectories in the US
