# Supplementary material for: Condensed trajectory of the temporal correlation of diseases and mortality extracted from over 300,000 patients in hospitals
Source: PLoS One. 2021 Oct 5;16(10):e0257894. doi: 10.1371/journal.pone.0257894 (PMC8491897; doi:10.1371/journal.pone.0257894)
Supplement: S2 Fig — We offers a clustered view of mortality trajectories by overlaid them by shared diagnoses and patterns of disease-to-disease progressions. (A-B) Of total 16 clusters, we present the first and second largest clusters. (A) The largest cluster, Cluster 12, covered disease patterns for >90,000 patients who had developed chronic obstructive pulmonary disease (COPD) and other circulatory heart diseases. (B) Cluster 7 depicts cancer and metastasis by tracking 17,781 patients and 1,566 deaths. Death nodes are hidden to improve the visibility of the clusters. (PDF) [file pone.0257894.s002.pdf]

A.

Cluster 12*, COPDs
(Chronic obstructive pulmonary diseases)

No. of deaths: 16,678
No. of patients: 90,965
No. of disease progressions: 50,183

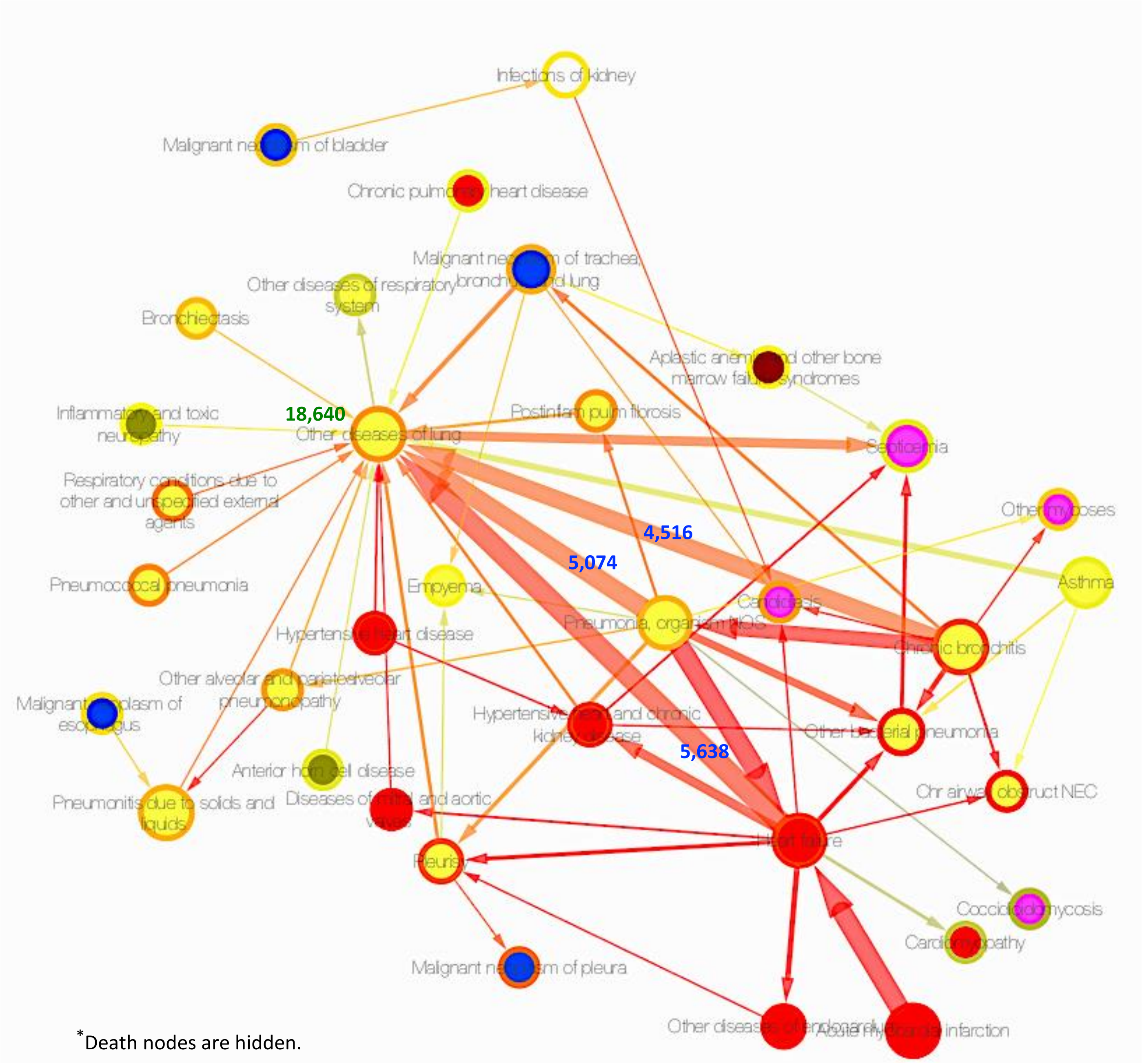

*Death nodes are hidden.

Supplemental figure 2. Cluster of trajectories

B.

Cluster 7*, Cancer & Metastases

No. of deaths: 1,566
No. of patients: 17,781
No. of disease progressions: 4,203

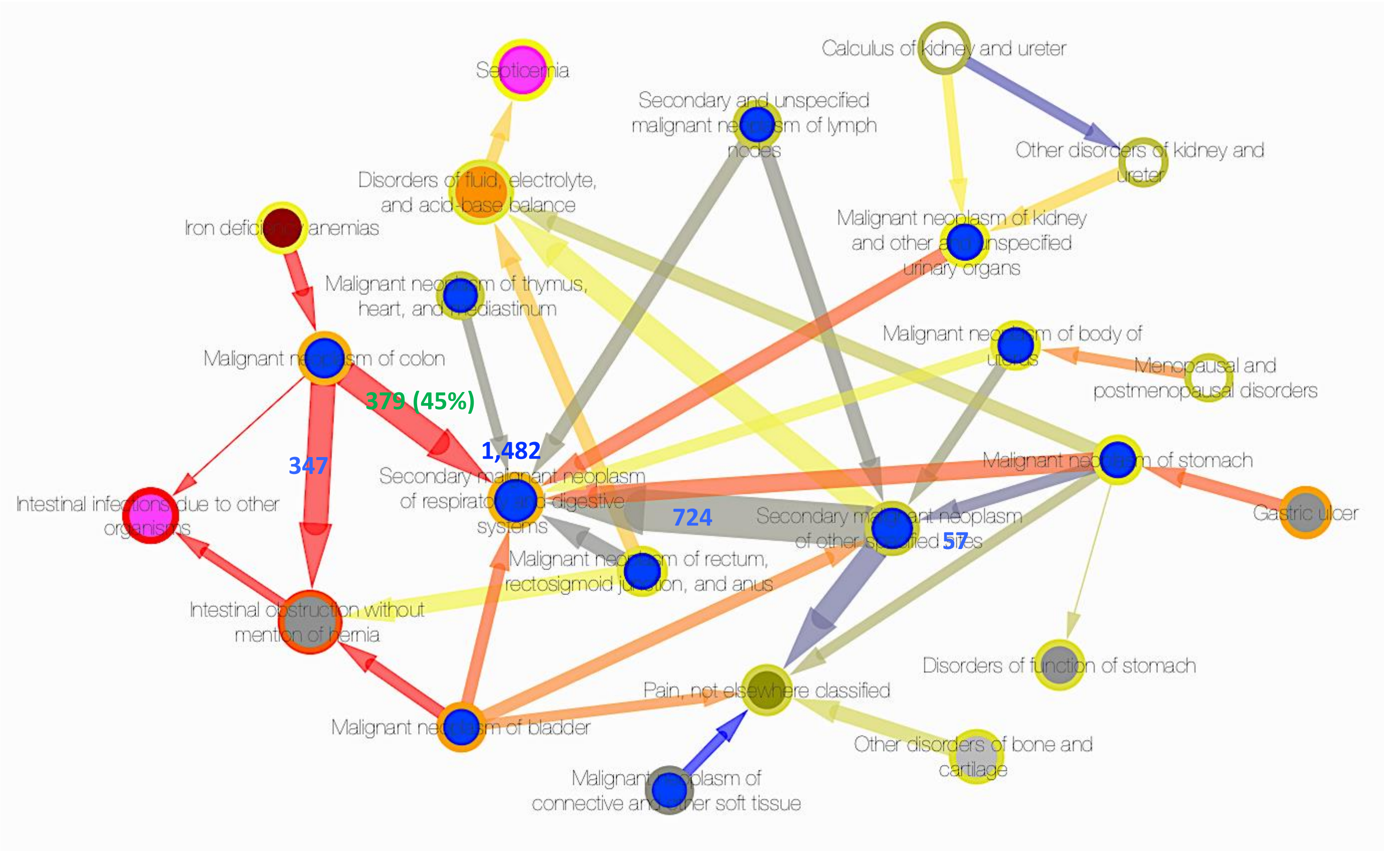

Disease (size by incidence)

Temporal disease progression (< 1year++)

<Color of node border and edge>

Mean age of patients

0~20 50 75

* Death nodes are hidden.
++ co-occurrences & temporal progressions of diseases (FDR <0.1)

<Node colors: types of diseases>

- Infectious and parasitic diseases
- Disease of respiratory system
- Disease of blood and blood forming organs
- Disease of circulatory system
- Disease of digestive system
- Diseases of musculoskeletal & connective tissue
- Disease of genitourinary system
- Neoplasms
- Endocrine, nutritional, metabolic & immune disease
- Disease of nervous system & sense organs

Supplemental figure 2. Cluster of trajectories
