# Supplementary material for: Condensed trajectory of the temporal correlation of diseases and mortality extracted from over 300,000 patients in hospitals
Source: PLoS One. 2021 Oct 5;16(10):e0257894. doi: 10.1371/journal.pone.0257894 (PMC8491897; doi:10.1371/journal.pone.0257894)
Supplement: S1 Table — Data resource of the Healthcare Cost and Utilization Project (HCUP) covering 97% of hospitals in the USA. 1Years of data set generations. Merged data set covers up to ~26.1 years of longitudinal events for a patient counted by administration month. For each inpatient event, up to 25 diagnosis codes were assigned. 2Covered years of records by build year versions. 3Excluded by diagnosis chapters for injury, symptom, childbirth, pregnancy, and healthcare service. (DOCX) [file pone.0257894.s003.docx]

**Supplemental Table 1.** Data statistics of the State Inpatient Database of California (SIDCA)^*^

| Features | No. recorded (in selected set)^3^ |
| --- | --- |
| No. of admissions  Data build in 2006^1^ (1980–2006)^2^  Data build in 2007^1^ (1988–2007)^2^  Data build in 2008^1^ (1987–2008)^2^  Data build in 2009^1^ (1988–2009)^2^  Data build in 2010^1^ (1980–2010)^2^ | 19,984,041 (**2,272,018)**  3,997,182 (128,516)  4,012,774 (126,768)  4,017,998 (124,632)  3,985,166 (121,661)  3,970,921 (1,770,441) |
| Grand total no. of patients  Data build in 2006  Data build in 2007  Data build in 2008  Data build in 2009  Data build in 2010 | 10,408,641 (**1,488,551)**  2,095,319 (68,237)  2,069,813 (66,611)  2,080,984 (66,048)  2,077,376 (63,691)  2,085,149 (1,223,964) |
| No. of unique diagnosis codes (ICD-9-CM)  No. of unique 3-digit diagnosis codes | 5,777  691 |
| Demographic features (in selected set)  Mean of age in the admission month  Gender  Outcomes of admission  Deaths  Survival | 63.77 (±19.58) years  M: 691,452 (46.4%), F: 780,230 (52.4%)  290,253 (19.5%)  1,334,635 |
